# Interaction of *Biomphalaria* cells in primary cultures with *Schistosoma mansoni* sporocysts

**DOI:** 10.1590/0037-8682-0257-2020

**Published:** 2020-11-06

**Authors:** Aristeu Silva-Neto, Cristhiane Oliveira da Fonseca, Luciana Maria Silva, Paulo Marcos Zech Coelho, Consuelo Latorre Fortes-Dias

**Affiliations:** 1 Fundação Oswaldo Cruz, Instituto René Rachou, Belo Horizonte, MG, Brasil.; 2 Fundação Ezequiel Dias, Diretoria de Pesquisa e Desenvolvimento, Belo Horizonte, MG, Brasil.

**Keywords:** Biomphalaria tenagophila, Biomphalaria glabrata, Schistosoma mansoni, Digestive gland, Saccular kidney, Primary cell culture

## Abstract

**INTRODUCTION:**

*Biomphalaria* snails may display varying levels of susceptibility to *Schistosoma mansoni* infection. We have been developing an *in vitro* model to study the interaction between the snail and the parasite, using tissue-derived cell cultures from *Biomphalaria*.

**METHODS:**

The digestive gland- and kidney-derived cells from primary cultures of resistant (*B. tenagophila* Taim) and susceptible (*B. tenagophila* HM and *B. glabrata* BH) strains of *Biomphalaria* were exposed to *S. mansoni* sporocysts.

**RESULTS:**

*S. mansoni* sporocysts were surrounded and encapsulated exclusively by cells derived from the digestive gland (DG) of *B. tenagophila* Taim. The process was followed by a marked decrease in the number of free sporocysts in the culture medium. The morphological characteristics of DG-derived cells in culture have been described.

**CONCLUSIONS:**

Cells derived from DG (but not SK) primary cultures of *B. tenagophila* Taim may participate in *S. mansoni* sporocyst control.

## INTRODUCTION

Schistosomiasis is a parasitic disease with a high prevalence in tropical and subtropical countries. In Brazil, 425,231 cases were recorded between 2008 and 2016, with the majority of cases in the northeastern (74.7%) and southeastern (24.8%) regions. In the same period, the infection positivity rate decreased from 5.28% (2008) to 3.40% (2016). However, the reduction in the percentage of positive cases should be analyzed with caution, since the number of people examined has also been decreasing every year, due to the difficulty in accessing testing services, the characteristics of the chronic disease, and underreporting of cases^1^. Owing to the low parasite load of the patients, only 25%-30% of those infected will be detected in areas of low endemicity for schistosomiasis. Since this is the case with the vast majority of endemic municipalities, it is estimated that the prevalence of schistosomiasis in Brazil is 2 to 3 times higher than that reported^2^.

The etiological agent of schistosomiasis in Latin America is the blood fluke *Schistosoma mansoni* Sambon 1907*,* which requires an essential passage through freshwater *Biomphalaria* (Preston, 1910) planorbids as part of its life cycle. After the deposition of *S. mansoni* eggs in water by an infected vertebrate, miracidia hatch and actively penetrate the tegument of the invertebrate host and may subsequently infect it. The snail’s internal defense system (IDS) has emerged as an important determinant of the success of *S. mansoni* infection, acting in concert with host and parasite genetics and epigenetics, proteomic and transcriptomic regulation, and environmental factors^3^. The IDS of *Biomphalaria* comprises circulating hemocytes and soluble factors contained in the hemolymph, which act together to eliminate infectious threats^4^.

In the literature, most studies of snail-trematode interactions utilize *Biomphalaria glabrata*-*S. mansoni* host-parasite model^3^ ^,^ ^5^. Genetic studies have demonstrated a spectrum of infection susceptibility depending on the host-parasite combination, and genetically selected susceptible (M-line and NMRI) or resistant (BS-90, 13-16-RI, and 10-R2) *B. glabrata* strains have become the model of choice for investigation of the mechanisms supporting resistance to *S. mansoni* infection^5^ ^,^ ^6^.

In Brazil, among the 3 species of medical importance in schistosomiasis-*B. glabrata* (Say, 1818), *B. straminea* (Dunker, 1848), and *B. tenagophila* (d’Orbigny, 1835)^7^-*B. tenagophila* is particularly interesting due to the existence of a naturally occurring strain completely resistant to infection by different strains (LE and SJ) of *S. mansoni* ^8^ ^,^ ^9^ **.** The resistant strain of *B. tenagophila* was originally isolated in the Ecological Reserve of Taim (Rio Grande do Sul state, Brazil) and named *B. tenagophila* Taim (BtT).

In the last few years, BtT has been used as our experimental model to investigate its interaction with *S. mansoni* using tissue-derived cell cultures. Successful primary cultures of the amebocyte-producing region (APO) were shown to be capable of eliminating *S. mansoni* sporocysts *in vitro* ^10^ ^,^ ^11^. Cells in primary cultures from the saccular portion of the kidney (SK) of BtT were indistinguishable from those obtained in APO-derived cultures^12^. Based on the cell resemblance and anatomical proximity of APO and kidney inside the mantle cavity of *Biomphalaria*, we suggested that primary cultures of kidney-derived cells from BtT might participate in hematopoiesis and in host response to parasitic invasion^12^. In the present study, the hypothesis was tested by challenging cultured cells derived from the SK of BtT with *S. mansoni* sporocysts. Tissue-derived cells from the digestive gland (DG) of the same strain, as well as SK- and DG-derived cell cultures from strains susceptible to *S. mansoni* infection, were used as experimental controls.

## METHODS

### Primary cell cultures from *Biomphalaria* tissues 

**FIGURE 1:**
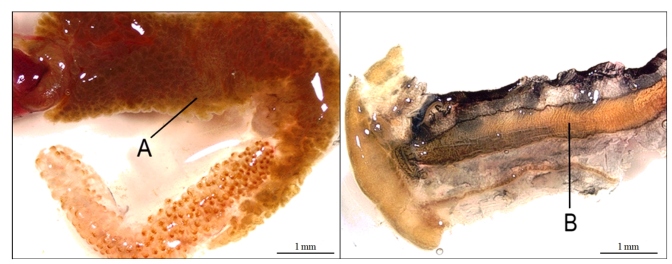
Dissected regions of *B. tenagophila* Taim (fresh snail) for explant preparation. **A.** Digestive gland (DG). **B.** Saccular portion of kidney (SK).

*Biomphalaria* specimens were provided by Instituto René Rachou. The number of specimens used per experiment varied according to the strain availability. In general, 10-20 adult individuals from each species or strain, with shell diameters of 10-18 mm, were submerged in 500 mL of metronidazole (250 mg/L) for 2 days, with one change of an equal volume of fresh solution in between. After anesthesia with sodium pentobarbital (0.4 mg/mL) for a minimum of 6 h, the shells were cleaned with 70% ethanol and broken between two glass slides. Individual tissue dissection was performed using surgical scissors under a stereo microscope (Stemi 200C, Carl Zeiss), previously cleaned with 70% ethanol, and subjected to UV light. The whole procedure was performed in a laminar flow hood using sterile reagents and materials. DGs and SKs (Figure 1) were excised, placed in glass plates and washed three times with Chernin's balanced saline solution (CBSS) consisting of 47.7 mM NaCl, 2 mM KCl, 0.49 mM Na_2_HPO_4_, 1.8 mM MgSO_4_∙7 H_2_O, 3.6 mM CaCl_2_∙2H_2_O, 0.59 mM NaHCO_3_, 5.5 mM glucose, and 3 mM trehalose, pH 7.4. Excisions of the same tissue from different specimens of the same strain were pooled together and sliced into 1 mm fragments. Two to three fragments from each pooled tissue were transferred to 12-well culture plates (Corning Costar, cat. 07-200-82) containing 500 μL/well of Schneider’s insect medium (Sigma-Aldrich, cat. S9895), and antibiotics (gentamicin (25 µg/mL), streptomycin (25 µg/mL), amphotericin B (10 µg/mL), and primocin (125 µg/mL)). The number of wells per experiment depended on the total amount of tissue fragments obtained in the particular experiment. The cultures were maintained at 26°C for 24 h in a humid chamber for maximum cell detachment and release into the medium. The wells were examined macroscopically and microscopically. Contaminated wells were discarded immediately. Cell viability was evaluated in sampled wells (n = 6) using the Trypan Blue method. A minimum of 90% cell viability was confirmed in each experiment.

Distinct aliquots of the cells from the DG of BtT were taken for phase contrast microscopic examination (Axiovert 200, Carl Zeiss) and after Giemsa (Merck Millipore) staining. Before staining, 300 µL of the cells in suspension were concentrated by centrifugation for 5 min at 1000 rpm. The supernatant was discarded, and the pellet containing the cells was resuspended in 600 μL of methanol and fixed onto glass slides. The fixed cells were stained and examined under a light field microscope (Axio Imager.A2, Carl Zeiss).

###  Preparation of *Schistosoma mansoni* sporocysts 

*In vitro*-transformed *S. mansoni* sporocysts from the SJ strain were obtained as described^13^. After transformation, the sporocyst-containing solution was concentrated by centrifugation at 810 rpm for 5 min, and the pellet was resuspended in 1 mL of CBSS (sporocyst stock solution). The sporocyst concentration in the stock solution was estimated by counting using a Neubauer chamber. *S. mansoni* sporocysts from the LE strain were prepared using the same procedure. Sporocyst viabilities were assessed using the Trypan Blue method. A minimum of 90% viability was obtained in the preparations.

### Challenging primary *Biomphalaria* cultures with *S. mansoni* sporocysts

Small volumes (microliters) of the stock solution containing about 15 *S. mansoni* sporocysts were added per cultured well, which contained 1 × 10^4^ viable cells on average. A preliminary test showed that the sporocysts were unaffected by the medium used for tissue-derived cultures. The interaction process between tissue-derived cells from *Biomphalaria* and *S. mansoni* sporocysts was followed using an inverted microscope (Axiovert 200, Carl Zeiss) for up to 24 h. The number of non-encapsulated sporocysts, freely floating in the culture medium, was counted per well of the culture plates.

## RESULTS

**FIGURE 2:**
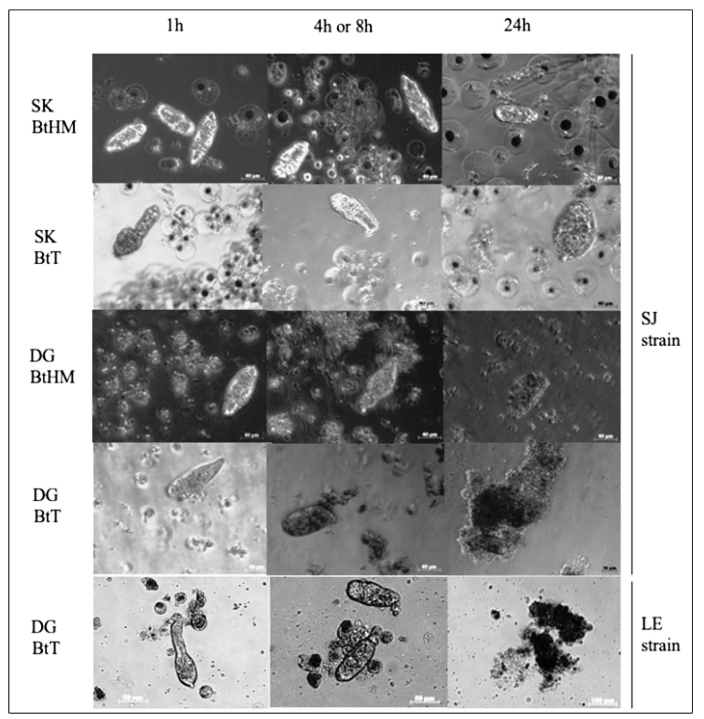
Photomicrographs of tissue-derived cells of the digestive gland (**DG**) and saccular portion of kidney (**SK**) from *B. tenagophila* Taim (**BtT**) and *B. tenagophila* Herivelton Martins (**BtHM**) after challenge with *S. mansoni* sporocysts from LE or SJ strain (indicated on the right side). Time after challenge is indicated on top.

After challenging the tissue-derived cultures with *S. mansoni* LE sporocysts, no detectable cell reaction was noted for SK or DG cultures obtained from susceptible BgBH (experimental control) strain, after up to 24 h of contact (data not shown). No cell reaction was observed in SK-derived cultures of the BtT (resistant) strain, as previously hypothesized. Unexpectedly, cells from the DG-derived cultures from BtT surrounded and completely encapsulated the sporocysts (Figure 2).

Since the susceptible *Biomphalaria* strain (BgBH) was from a different species, *B. glabrata* was replaced by a susceptible strain of *B. tenagophila* (BtHM). The challenge was performed with *S. mansoni* sporocysts from the SJ strain. No cell reaction was observed for either SK or DG tissues of BtHM in primary culture Again, DG-derived cells, but not SK-derived cells, from BtT surrounded and encapsulated *S. mansoni* sporocysts (Figure 2).

**FIGURE 3:**
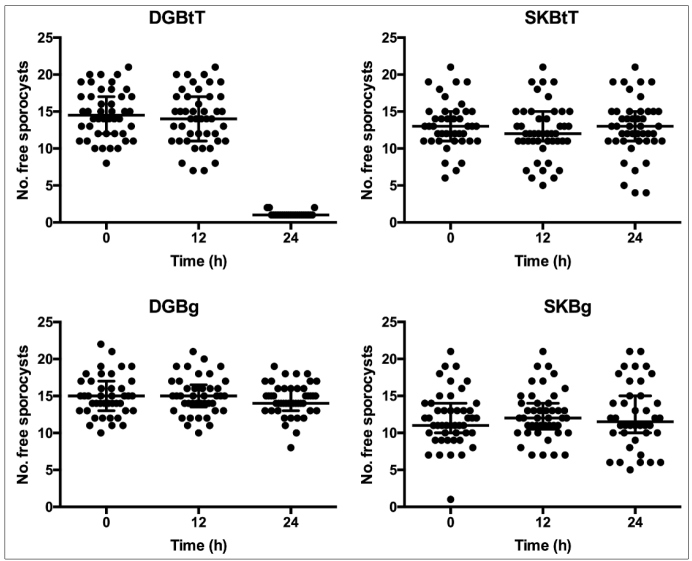
Timeline of the challenge of tissue-derived (**DG:** digestive gland; **SK:** saccular portion of kidney) cell cultures from *Biomphalaria* with *S. mansoni* sporocysts from the LE strain. Tissue origin: **DGBtT,** Digestive gland of *B. tenagophila* Taim; **SKBtT,** saccular portion of kidney of *B. tenagophila* Taim; **DGBg,** Digestive gland of *B. glabrata*; **SKBg,** saccular portion of kidney of *B. glabrata.* Each point represents a replicate, i.e., sporocyst counting per cell culture well prepared from a pooled sample of the respective tissues excised from 10-20 *Biomphalaria* specimens. Horizontal bars represent median and interquartile ranges for each group of data.

The number of free, non-encapsulated sporocysts was quantified in DG- and SK-derived cell cultures obtained from BgBH (susceptible) and BtT (resistant) strains. The exclusive action of DG-derived cells from BtT (resistant) was confirmed by a marked decrease in the number of free sporocysts (Figure 3).

**FIGURE 4:**
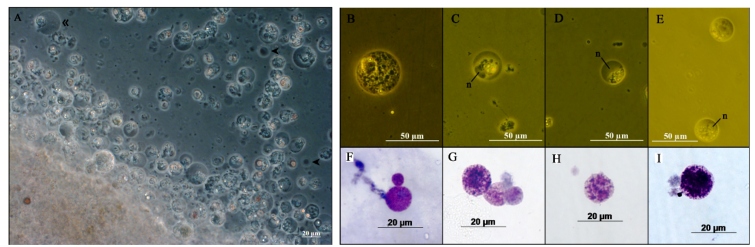
DG-derived cells from *B. tenagophila* Taim in primary culture. A: General view of the culture. A wide diversity of cells with different sizes and morphologies spontaneously detached from the tissue explant and migrated into the culture medium. Most of them are rounded shape, with eccentric nucleus and granulated cytoplasm. Cells resembling type I (marked by single head) and type II (marked by double head) cell populations from previous APO-derived culture were observed at minor amounts. **B-E:** Detailed view of some isolated cells from the culture. **B:** Rounded cell with fully granulated cytoplasm. **C:** Rounded cell with clustered granules adjacent to eccentric nucleus (n). **D:** Rounded cell with large, eccentric nucleus (n) and granulated cytoplasm resembling granulocytes described in *Biomphalaria* hemolymph (see text). **E:** Rounded cell with eccentric nucleus (n) and granulated cytoplasm. **F-I:** Giemsa-stained cells with different ratios of acidophilic (pink color) and basophilic (purple) granules in the cytoplasm.

In an attempt to identify the cells responsible for the encapsulation, we examined the DG-derived cells from BtT in culture (Figure 4). Cells with diverse sizes and morphologies were observed migrating from the tissue explant to the culture medium (Figure 4A). Most of them contained granules and possible secreting vesicles, with different distribution profiles and eccentric nuclei. Among the cells in culture, four morphological types were isolated (Figure 4B-D). Giemsa staining showed the concomitant presence of acidophilic and basophilic granules in the cytoplasm of cells (Figure 4F-I).

**FIGURE 5:**
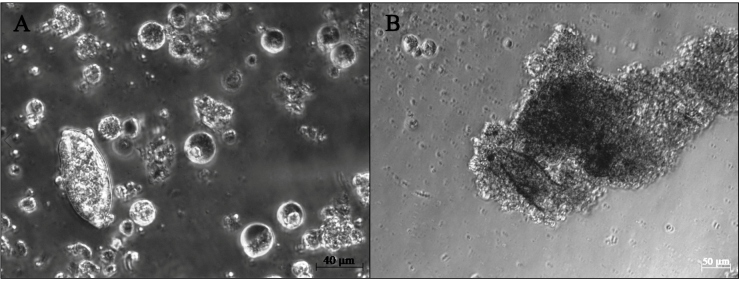
Challenge of DG-derived primary culture of *B. tenagophila* Taim with *S. mansoni* sporocysts. **A:** After 4 h of contact. The sporocyst is floating in the medium with no apparent reaction of the cells in culture. **B:** After 24 h of contact. Sporocyst(s) encapsulated by a tangled network of cells. A number of cell nuclei can be noted as black rounded spots in the network.

Figure 5 shows a closer view of the DG-derived culture from BtT after two different times (4 h and 24 h) of contact with *S. mansoni*. After 4 h of contact (Figure 5A), there was no apparent reaction of the cells against the sporocyst. Twenty hours later, the sporocyst was encapsulated by cells (Figure 5B). Although some nuclei were visible, it was impossible to identify which cell type(s) they belong to in the tangled network of cells.

## DISCUSSION

All the cells in the tissue-derived cultures from *Biomphalaria* detached and migrated to the culture medium without the need for any cell dispersion treatment. Similar behavior was observed for the primary cultures of the amebocyte-producing organ (APO) from BtT, except that the authors had used a CRML 1415 culture medium with supplements, including fetal bovine serum^10^. The culture medium was replaced with Schneider’s insect medium and the cultures were maintained at 26°C. The condition ensured at least 90% cell viability after 36 h in culture^12^.

Hemocytes are critical in the *Biomphalaria* response to an invading schistosome as primary immune effector cells comprising the host’s IDS. With variable morphology and enzymatic content, the hemocytes display different adhesion and phagocytosis properties. In resistant *Biomphalaria* strains, hemocytes surround, rapidly encapsulate, and destroy the parasite. Proteomic analysis of hemocytes from susceptible (NMRI) and resistant (BS-90) strains of *B. glabrata,* exposed or not exposed to *S. mansoni* sporocysts, was performed. The study revealed a unique expression profile of antimicrobial/antiparasitic proteins in the resistant strain (BS-90) during *in vitro* encapsulation of the parasite. Differences in the expression profiles of immune-related proteins and those involved in protein/CHO metabolism, redox pathways, and signaling pathways were also noted between the susceptible and resistant strains^14^. Without the hemocyte-driven encapsulation response, the parasite often survives and may establish infection in the host. Hemocyte activation triggers the production of cytotoxic molecules, the best known being reactive oxygen species^3^. *In vivo* experiments with *B. tenagophila* Taim post *S. mansoni* infection showed damaged sporocysts inside the resistant snails, with complete destruction of the characteristic structures of the parasite after 10 h^11^. Sporocyst morphology was not affected in the susceptible *B. tenagophila* (Cabo Frio) strain. Sporocyst killing was time-dependent and exclusive to hemocytes from the resistant strain. In the present study, sporocysts were not isolated from the cultures for morphological analysis or viability assessment. We cannot discard the possibility that cell-released factors may have started the killing of free sporocysts at 12 h and eventually resulted in encapsulation of already dead sporocysts after 24 h. However, cell migration and subsequent encapsulation of *S. mansoni* sporocysts is known to be the first step towards parasite killing. In our *in vitro* model, the behavior was exclusively observed for DG-derived cells from the BtT (resistant) strain. The lack of any cell reaction in SK-derived cultures from BtT failed to prove the previous hypothesis that primary cultures from the tissue might participate in hematopoiesis and invertebrate host response to *S. mansoni* infection^12^.

There is no consensus in the literature on the classification of hemocytes. In the past, a number of authors reported the presence of two subpopulations of hemocytes in *Biomphalaria*, the so-called granulocytes (representing 90% of the circulating cells) and hyalinocytes^4^ ^,^ ^15^ ^,^ ^16^. More recently, 5 morphological types have been described in the hemolymph of *B. glabrata* and *B. straminea*: blast-like cells, granulocytes, type I hyalinocytes, type II hyalinocytes, and type III hyalinocytes^17^. The main feature of blast-like cells was a large centrally located nucleus that almost filled the whole cell. According to the authors, they represented approximately 45% of the total hemocyte population in the hemolymph. Granulocytes, which were filled with a variable number of basophilic granules, were identified at minor percentages (4%-5% of the total cell population). The plasma membrane of type I hyalinocytes was irregular, with cytoplasm projections-filopodia and pseudopodia-whereas types II and III were oval-shaped with more homogeneous cytoplasm. On the other hand, based mostly on the nuclei-to-cytoplasm ratios, 3 cell populations (called I, II, and III) were identified in primary cultures of the APO^10^. Type I comprised the predominant cell population in culture, with a higher nucleus-to-cytoplasm ratio and morphologically similar to hyalinocytes of the hemolymph^4^. Type II cells displayed a relatively lower nucleus-to-cytoplasm ratio than type I cells and were compared to granulocytes^4^. At that time, a single cell type that resembled subpopulation II in the primary cultures was identified in the hemolymph of *B. glabrata*. The last cell population in culture, named III, was characteristically light-refringent under phase-contrast microscopy, with no defined nuclei.

In the present study, a wide diversity of cells was observed in the DG-derived cultures (Figure 4A). The majority of cells in culture displayed cytoplasmic granulations (Figure 4A-E), which is consistent with the expected profile for secretory gland tissues^12^. In Figure 4A, it is possible to identify the presence of cells with apparent morphological resemblance to Type I or Type II cell populations from the APO cultures^10^. However, these kind of cells (indicated in Figure 4A) were observed at very low numbers in the DG-derived culture and were not isolated for a more detailed examination. Cells similar to granulocytes from *Biomphalaria* hemolymph^17^, exhibiting large eccentric nuclei and granulated cytoplasm shifted to the periphery, were also noted (Figure 4D). The DG-derived cells displayed the concomitant presence of basophilic and acidophilic granules (Figure 4F-I). Cytoplasmic projections were not observed for any cell in culture. The diversity of cell types in the DG-derived cultures and the tangled cell network that surrounded the sporocyst after 24 h hindered any specific identification of the cell(s) responsible for the phenomenon (Figure 5).

The site of origin of hemocytes circulating in the hemolymph or fixed in tissues is also a subject of discussion in the literature^3^ ^,^ ^18^ ^,^ ^19^. There are indications that APO is the site of hemocyte production, with a function similar to that of bone marrow in vertebrates^20^ ^-^ ^22^. More recently, however, it has been shown that hemocytes can be formed in any organ and may originate from cells lining the venous sinuses or cells situated within loose connective tissue^19^. Although the tissues used for the primary cultures were washed three times before fragmentation, it is important to consider that snails have an open circulatory system and tissues are filled with hemolymph. Contamination of cultures with residual circulating hemolymph or resident hemocytes^4^ cannot be disregarded. Since we did not isolate the cells from encapsulated sporocysts, we have no evidence to suggest which type of cell(s) would be the effector cell(s). Regarding the origin of the cells, our data do not support any assumptions. They might have originated from resident hemocytes or from any type of specific cell in the tissue. It is possible that the culture conditions stimulated the cells to secrete soluble factors, similar to those present in the hemolymph, which might have stimulated cell differentiation. However, in two strains of *B. glabrata* (13-16-R1 and 10-R2) that are constitutively resistant to *S. mansoni* infection, hemocytes in plasma-free conditions were able to kill sporocysts as effectively as whole hemolymph^23^. The authors concluded that humoral factors played a role in *Biomphalaria*’s IDS, but they were not absolutely required for the ability to kill parasites. It was suggested that there were intrinsic differences between hemocytes present in susceptible and resistant strains.

Studies performed in the last 30 years have suggested that BtT may be used as a tool for the biological control of schistosomiasis transmission. Field trials of the physical introduction of BtT into a stream containing only BtHM (susceptible) are promising. Susceptibility of snail offspring to *S. mansoni* infection decreased from 38.6% to 2.1% fourteen months after the introduction. A significant correlation was observed between the absence of infection and the presence of an exclusive 350 bp genetic marker for BtT in the offspring^24^.

In conclusion, our data reinforce the applicability of *in vitro* models, such as tissue-derived primary cultures, to investigate the interaction between *Biomphalaria* and *S. mansoni* and the mechanisms underlying susceptibility or resistance of the invertebrate host to parasite infection. Further isolation and characterization of DG-derived cells are needed to clarify the mechanism involved in their response to invading *S. mansoni* sporocysts in resistant *Biomphalaria* strains.
